# A Field Test of Web-Based Screening for Dry Eye Disease to Enhance Awareness of Eye Problems Among General Internet Users: A Latent Strategy to Promote Health

**DOI:** 10.2196/jmir.2198

**Published:** 2013-09-27

**Authors:** Motoko Kawashima, Miki Uchino, Takashi Kawazoe, Masaaki Kamiyashiki, Kokoro Sano, Kazuo Tsubota

**Affiliations:** ^1^Department of OphthalomolgyKeio University School of MedicineShinjuku-kuJapan; ^2^Ryogoku Eye ClinicSumida-kuJapan; ^3^Carepro IncShibuya-kuJapan

**Keywords:** dry eye, Schirmer test, tears, Internet, questionnaire, screening, Web, health care

## Abstract

**Background:**

A Web-based self-check system including a brief questionnaire would seem to be a suitable tool for rapid disease screening.

**Objective:**

The purpose of this preliminary study was to test a Web-based self-screening questionnaire for drawing attention to dry eye disease among general Internet users and identifying those with a higher risk of developing the condition.

**Methods:**

A survey website was launched and used to recruit participants from general Internet users. In the first phase, volunteers were asked to complete a Web-based self-screening questionnaire containing 12 questions on dry eye symptoms. The second phase focused on the respondents who reported five or more dry eye symptoms and expressed their intention to seek medical attention. These participants performed the Schirmer test, for evaluating tear production, and completed a paper-based lifestyle questionnaire to provide relevant background data.

**Results:**

Of the 1689 visitors to the website, 980 (58.0%) volunteers completed the Web-based self-screening questionnaire. Among these, 355 (36.2%) respondents reported five or more dry eye symptoms. Then, 99 (27.9%) of the symptomatic participants performed the Schirmer test and completed the paper-based lifestyle questionnaire. Out of these, 32 (32.2%) had abnormal tear production (≤5 mm).

**Conclusions:**

The proposed Web-based self-screening questionnaire seems to be a promising tool for raising awareness of dry eye disease among general Internet users and identifying those with a higher risk of developing the condition, although further research is needed to validate its effectiveness.

## Introduction

Dry eye syndrome (International Classification of Diseases, 10th revision, clinical modification code H04.1) is a highly prevalent chronic ocular-surface disease [1-14]. According to the International Dry Eye Workshop report, it can be defined as a multifactorial disease of the tears and ocular surface that results in symptoms of discomfort, visual disturbance, and tear-film instability with potential damage to the ocular surface [1]. The most common symptoms are dry eye sensation, foreign body sensation, photophobia, and red eye. These symptoms can be debilitating and cause difficulties in visual functioning and other tasks requiring sustained visual concentration [15-23]. They can also have a negative impact on physical, social, and psychologic health and the overall sense of well-being [15-23].

The increasing prevalence of dry eye disease worldwide is an important public health problem, especially in developed countries with advanced information technology and those with an aging population [1-14]. In Japan, the incidence rates of dry eye disease are almost 22% and are over 10% in female and male computer users, respectively, implying that the country has more than 24 million affected individuals [2]. One reason for the rapid rise in the number of cases of dry eye disease over the last few years is thought to be prolonged visual display terminal (VDT) exposure because of increased computer use. VDT exposure is also becoming common in the general population because of the widespread use of mobile technology and portable information terminals, especially smartphones, among all age groups. The number of Internet users worldwide has doubled in the past 5 years. Japan had approximately 94 million Internet users in 2010, representing 78.2% of the Japanese population [24].

These changes in work and leisure activities have been accompanied by an increase in the number of reported symptoms of several health problems associated with VDT use. Eye problems, constituting a widespread but largely unknown epidemic among computer users, are the most common symptoms [25-30]. Dry eye is a common cause of, or is at least associated with, greater asthenopia symptoms among VDT workers [31-35]. Clinical observations have long suggested that VDT users may be at increased risk of developing dry eye disease [2,15,36], and epidemiologic data evidence the magnitude of the problem [36-38]. In a recent clinical study, the prevalence rate of definite dry eye disease was 8.0% and 18.7% in male and female VDT users, respectively, and that of probable dry eye disease was 52.1% and 57.8%, respectively [3], supporting the view that VDT exposure is a significant risk factor of this condition.

A clinical diagnosis of dry eye disease requires both objective findings from eye examinations and a subjective report of dry eye symptoms. In several studies, a subjective report of the symptoms was the sole criterion for the diagnosis of dry eye disease [2,4-10]; this diagnostic basis was considered appropriate because the condition rarely progresses to the stage of ocular discomfort without symptom presentation [1,20]. However, general Internet and heavy VDT users may be unaware of dry eye disease despite the presence of symptoms. Another problem is that many individuals with dry eye disease do not receive medical intervention [21,22].

In this regard, a Web-based questionnaire targeting VDT users as a high-risk population would be effective. The World Wide Web has enabled the increased use of questionnaires to collect data for various research surveys [39-41]. This tool can be optimized to promote awareness of dry eye disease. However, until now, Web-based self-screening of dry eye disease has not been attempted.

The purpose of this study was to test a Web-based self-screening questionnaire for drawing attention to dry eye disease among general Internet users and identifying those with a higher risk of developing the condition.

## Methods

### Study Design

#### Phases

This preliminary study consisted of two phases: the first phase involved a Web-based survey with a self-screening questionnaire on dry eye symptoms, and the second phase involved measurement of tear secretion and a physical survey with a paper-based lifestyle questionnaire. The volunteers were free to participate in either the first phase only or both phases of the study. The study duration was from September 1 to December 29, 2011.

#### Ethical Statement

The research was conducted in accordance with the ethical principles of the Declaration of Helsinki and was based on a protocol approved by the Institutional Review Board of Ryogoku Eye Clinic. Written informed consent, including approval for the use of information collected during the study, was obtained from the participants through the survey website.

### First Phase

#### Survey Website

In this phase, an informative survey website was first launched. Then, location-specific advertising banners were placed on different websites and search engines by employing Google AdSense to recruit a cross-section of prospective subjects from general Internet users. Because the second phase of the study was to be conducted in Yokohama, Kanagawa Prefecture, volunteers with easy access to Yokohama were preferred. Study information was provided via tweets and posts on Facebook. Every visit to the survey website was recorded. Underage visitors and those receiving medical treatment for any eye problems were excluded. Registration was free, and the volunteers received no compensation for participation.

#### Web-Based Self-Screening Questionnaire

**Figure 1:**
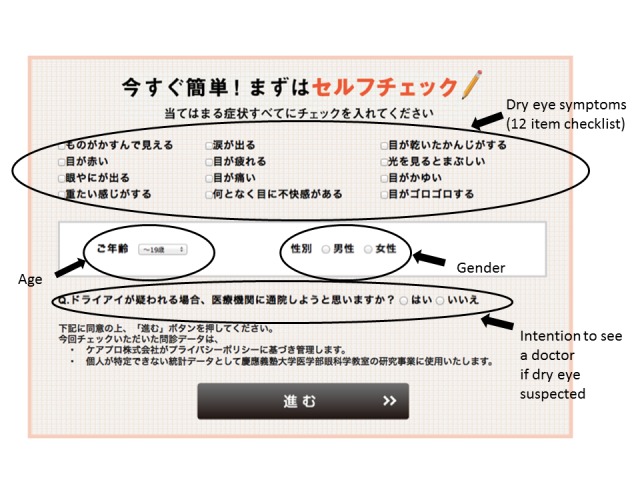
Web-based self-screening questionnaire.

The applied Web-based self-screening questionnaire was a modified version of the questionnaire used by Toda et al [31], which is generally used for clinical diagnosis of dry eye disease in Japan. The questionnaire consisted of 12 questions regarding dry eye symptoms, and only “yes” or “no” responses via checkboxes were allowed (Figure 1). Respondents with five or more dry eye symptoms were considered to have subjective dry eye symptomatology.

All the respondents were requested to provide an email address voluntarily. Those with subjective dry eye symptomatology were encouraged to participate in further tests and asked about their intention to see a doctor for definitive diagnosis, to assess whether the online self-check system could motivate Internet users to seek medical attention and because they were considered to have a higher risk of dry eye disease.

The following data were also collected: number of visitors to the website, number of respondents to the questionnaire, and descriptive data of the participants (age group, gender, and symptom distribution).

### Second Phase

#### Overview

The second phase of the study was directed at the respondents with subjective dry eye symptomatology, who gave their contact information and consent for the study. The research site was a health center in Yokohama, and only nurses were present.

#### Schirmer Test

Tear production was measured by using the Schirmer test [42], which is the most common objective diagnostic test for dry eye disease. The participants of this phase performed the test without anesthesia (Schirmer method I) by themselves under the nurses’ instructions, as is allowed in Japan. The procedure was approved by the local public health center. The participants with tear production of ≤5 mm were advised to see a doctor (at any clinic of their choice).

#### Paper-Based Lifestyle Questionnaire

This questionnaire included items on the durations of VDT and contact lens use, because these are the major factors contributing to dry eye disease [1-3,36]. To collect relevant background data, health-related physical activity was also examined by using the International Physical Activity Questionnaire (IPAQ) [43,44]. Each participant’s physical activity level in metabolic equivalent (MET) per week (MET, min/week) was then calculated.

### Statistical Analysis

Data were analyzed by using JMP statistical discovery software version 9.0 (SAS Institute). Independent-sample *t* tests were used to determine whether the parametric differences between those with and without an intention to seek a medical opinion for suspected dry eye disease, as well as between those with >5 mm (normal) and ≤5 mm (abnormal) tear production, were significant. *P<*.05 was considered significant.

## Results

### Characteristics of the First-Phase Participants

**Figure 2:**
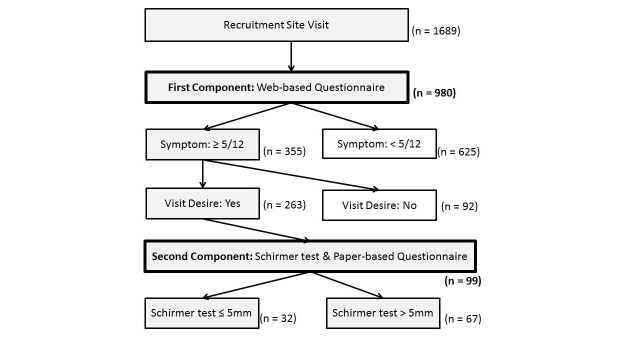
Flow diagram of participants.

Figure 2 depicts the flow diagram of the study. Of the 1689 visitors to the survey website, 980 (58.0%) volunteers, including 440 men (44.9%), 539 women (55.1%), and one person of unidentified gender, completed the Web-based self-screening questionnaire. Their demographic data are summarized in Table 1. Ocular fatigue (61.4%), dry eye sensation (40.7%), and sensitivity to bright light (31.3%) were the major symptoms.

Of the 979 gender-identified respondents, 355 participants had subjective dry eye symptomatology. The symptoms were significantly more common among women than among men (226 female participants, 63.7%, *P*=.0001, Fisher’s exact test). Ninety-two participants (25.9%) did not intend to seek medical attention for the symptoms (Table 2). Men were significantly less inclined to visit a clinic for definitive diagnosis (*P=*.006, Fisher’s exact test). Further, significantly fewer participants in their 20s intended to see a doctor (*P<*.001, chi-square test).

**Table 1:** Characteristics of the 980 first-phase participants.

Characteristic	Category	Total, n (%)
**Gender**		
	Male	440 (44.9)
	Female	539 (55.1)
	Unidentified	1
**Age group, years**		
	20–29	446 (45.5)
	30–39	227 (23.2)
	40–49	158 (16.1)
	50–59	73 (7.4)
	>60	76 (7.8)
**Dry eye symptom**		
	Ocular fatigue	602 (61.4)
	Discharge	296 (30.2)
	Foreign body sensation	227 (23.2)
	Heavy sensation	213 (21.7)
	Dry sensation	399 (40.7)
	Uncomfortable sensation	271 (27.7)
	Excess tearing	166 (16.9)
	Blurred vision	296 (30.2)
	Itching	252 (25.7)
	Sensitivity to bright light	307 (31.3)
	Redness	198 (20.2)
	Pain	189 (19.3)

**Table 2:** Characteristics of the 979 gender-identified respondents^a^.

Characteristic	Category	“Yes” response, n (%)	“No” response, n (%)	*P*
**Number of dry eye symptoms**				
	<5	428 (68.6)	196 (31.4)	.080^b^
	≥5	263 (74.1)	92 (25.9)	
**Gender**				
	Male	291 (66.1)	149 (33.9)	.006^b^
	Female	400 (74.2)	139 (25.8)	
**Age group, years**				
	20–29	291 (65.4)	154 (34.6)	.004^c^
	30–39	160 (70.5)	67 (29.5)	
	40–49	120 (75.9)	38 (24.1)	
	50–59	60 (82.2)	13 (17.8)	
	>60	60 (78.9	16 (21.1)	

^a^One of the 980 respondents did not provide information about gender.

^b^Fisher’s exact test.

^c^Chi-square test.

### Characteristics of the Second-Phase Participants

Of the 355 participants with subjective dry eye symptomatology, 99 (27.9%) participants, including 24 men (24%) and 75 women (76%), performed the Schirmer test and answered the paper-based lifestyle questionnaire. Most of these participants were healthy: only 19.2% had a systemic disease. Contact lenses were used by 40.4%, and 10.1% had a smoking history (Table 3).

Thirty-two participants had tear production of ≤5 mm (Table 4). No significant differences were noted in the duration of VDT use (*P=*.82, Wilcoxon rank-sum test), use of contact lenses (*P=*.67, Fisher’s exact test), duration of contact lens use (*P=*.98, *t* test), and smoking history (*P=*.49, Fisher’s exact test) between the >5 mm and ≤5 mm tear production groups. A significant difference was found only in exercise habits: a lower level of physical activity was significantly associated with a lower value in the Schirmer test (*P=*.02, Wilcoxon rank-sum test).

**Table 3:** Characteristics of the 99 second-phase participants.

Characteristic	Category	Total, n (%)
**Gender**		
	Male	24 (24)
	Female	75 (76)
**Age group, years**		
	20–29	29 (29.3)
	30–39	25 (25.2)
	40–49	14 (14.1)
	50–59	15 (15.2)
	>60	16 (16.2)
**Dry eye symptom**		
	Ocular fatigue	90 (90.9)
	Discharge	46 (46.5)
	Foreign body sensation	43 (43.4)
	Heavy sensation	52 (52.5)
	Dry sensation	66 (66.7)
	Uncomfortable sensation	66 (66.7)
	Excess tearing	21 (21.2)
	Blurred vision	53 (53.5)
	Itching	40 (41.7)
	Sensitivity to bright light	37 (37.4)
	Redness	38 (38.4)
	Pain	33 (33.3)
**Related lifestyle habits**		
	Duration of VDT use, mean hours (SD)	5.8 (3.0)
	Contact lens use	40 (40.4)
	Duration of contact lens use, mean hours (SD)	12.6 (3.2)
	Smoking	10 (10.1)
	Systemic disease	19 (19.2)

**Table 4:** Comparison of the groups with >5 mm (normal) and ≤5 mm (abnormal) tear production.

Characteristic	Category	>5 mm, n (%)	≤5 mm, n (%)	*P*
**Gender**				
	Male	18 (75.0)	6 (25.0)	.458^a^
	Female	49 (65.3)	26 (34.7)	
**Age group, years**				
	20–29	25 (86.2)	4 (13.8)	.022^b^
	30–39	18 (72.0)	7 (28.0)	
	40–49	6 (42.9)	8 (57.1)	
	50–59	8 (53.3)	7 (46.7)	
	>60	10 (62.5)	6 (37.5)	
Duration of VDT use, mean hours (SD)		5.9 (2.9)	5.9 (3.2)	.821^c^
Contact lens use		26 (38.8)	14 (43.8)	.667^a^
Duration of contact lens use, mean hours (SD)		12.6 (3.5)	12.6 (2.7)	.978^d^
Smoking		8 (11.2)	2 (6.3)	.493^a^
Systemic disease		10 (14.9)	9 (28.2)	.171^a^

^a^Fisher’s exact test.

^b^Chi-square test.

^c^Wilcoxon rank-sum test.

^d^ *t* test.

## Discussion

### Principal Results

In this study, a Web-based self-screening questionnaire was used to draw attention to dry eye disease among general Internet users and identify those at higher risk. Of the 1689 visitors to the survey website, 58.0% (980/1689) completed the first phase of the study, 36.2% (355/980) had five or more dry eye symptoms, 27.9% (99/355) completed the second phase of the study, and 32.3% (32/99) had tear production of ≤5 mm. This self-screening tool therefore identified a few individuals with probable dry eye disease. We speculate that a high number of general Internet users have latent dry eye symptomatology.

### Comparison With Previous Research

In the first phase of this study, 63.7% (226/355) of the female participants reported five or more dry eye symptoms, supporting previous conclusions that dry eye disease is more common in women [1-3,5-7,36].

Overall, the participants in phase 2 were generally healthy and health conscious, with a low percentage of smokers and good reported dietary habits (data not shown). A lower level of physical activity was significantly associated with a lower value in the Schirmer test. Notably, 25.9% of the 355 participants who had five or more dry eye symptoms, especially men and people in their 20s, did not intend to see a doctor. The self-screening questionnaire may have slightly improved their health consciousness but was not sufficient to persuade them to seek medical care. Therefore, only some VDT users may be motivated enough to visit a doctor despite having symptoms. Ophthalmologists should promote awareness of dry eye disease more proactively, and the public should be educated about the condition for early detection and intervention.

### Limitations and Future Research

First, because the participants were recruited from general Internet users in a limited target area, a considerable part of the high-risk population was excluded. The recruitment method would have also introduced selection bias. Second, the participants with fewer than five dry eye symptoms were excluded from the physical phase of the study. Therefore, parametric differences between these participants and the participants with subjective dry eye symptomatology were not analyzed. Third, the second-phase results were based on the Schirmer test, which measures tear production; therefore, the evaporative aspects of dry eye disease were overlooked. Finally, the Web-based self-screening questionnaire has not yet been validated; the validation process is now underway under the supervision of the Japan Dry Eye Research Society.

Future research should include both biologic measures (break-up time and fluorescein staining) and the Schirmer test to diagnose dry eye disease. In addition, a normal control group and general Internet users in a wider geographic area should be included. Moreover, the self-screening system needs to be improved. As the next research step, the Web-based self-screening questionnaire should be optimized and validated to ensure its effectiveness in increasing awareness of dry eye disease especially among VDT users.

### Conclusion

The proposed Web-based self-screening questionnaire seems to be a promising tool to raise awareness of dry eye disease among general Internet users and identify those with a higher risk of developing the condition. The factors that encourage people with probable dry eye symptomatology to seek medical help are yet to be identified. Further research is required to provide sufficient information on the disease and diagnostic tests, determine symptom severity via a validated self-screening tool, and introduce improved diagnostic measures.

## Abbreviations

IPAQ: International Physical Activity Questionnaire
MET: metabolic equivalent
VDT: visual display terminal

## References

[1] DEWS (2007). The definition and classification of dry eye disease: report of the Definition and Classification Subcommittee of the International Dry Eye WorkShop (2007). Ocul Surf.

[2] Uchino M, Schaumberg DA, Dogru M, Uchino Y, Fukagawa K, Shimmura S, Satoh T, Takebayashi T, Tsubota K (2008). Prevalence of dry eye disease among Japanese visual display terminal users. Ophthalmology.

[3] Uchino M, Yokoi N, Uchino Y, Dogru M, Kawashima M, Komuro A, Sonomura Y, Kato H, Kinoshita S, Schaumberg DA, Tsubota K (2013). Prevalence of Dry Eye Disease and its Risk Factors in Visual Display Terminal Users: The Osaka Study. Am J Ophthalmol.

[4] Doughty MJ, Fonn D, Richter D, Simpson T, Caffery B, Gordon K (1997). A patient questionnaire approach to estimating the prevalence of dry eye symptoms in patients presenting to optometric practices across Canada. Optom Vis Sci.

[5] Moss SE, Klein R, Klein BE (2000). Prevalence of and risk factors for dry eye syndrome. Arch Ophthalmol.

[6] Lee AJ, Lee J, Saw SM, Gazzard G, Koh D, Widjaja D, Tan DT (2002). Prevalence and risk factors associated with dry eye symptoms: a population based study in Indonesia. Br J Ophthalmol.

[7] Chia EM, Mitchell P, Rochtchina E, Lee AJ, Maroun R, Wang JJ (2003). Prevalence and associations of dry eye syndrome in an older population: the Blue Mountains Eye Study. Clin Experiment Ophthalmol.

[8] Begley CG, Caffery B, Nichols K, Mitchell GL, Chalmers R, DREI study group (2002). Results of a dry eye questionnaire from optometric practices in North America. Adv Exp Med Biol.

[9] Caffery BE, Richter D, Simpson T, Fonn D, Doughty M, Gordon K (1998). CANDEES. The Canadian Dry Eye Epidemiology Study. Adv Exp Med Biol.

[10] Schein OD, Muñoz B, Tielsch JM, Bandeen-Roche K, West S (1997). Prevalence of dry eye among the elderly. Am J Ophthalmol.

[11] McCarty CA, Bansal AK, Livingston PM, Stanislavsky YL, Taylor HR (1998). The epidemiology of dry eye in Melbourne, Australia. Ophthalmology.

[12] Schaumberg DA, Sullivan DA, Buring JE, Dana MR (2003). Prevalence of dry eye syndrome among US women. Am J Ophthalmol.

[13] Lin PY, Tsai SY, Cheng CY, Liu JH, Chou P, Hsu WM (2003). Prevalence of dry eye among an elderly Chinese population in Taiwan: the Shihpai Eye Study. Ophthalmology.

[14] DEWS (2007). The epidemiology of dry eye disease: report of the Epidemiology Subcommittee of the International Dry Eye WorkShop (2007). Ocul Surf.

[15] Miljanović B, Dana R, Sullivan DA, Schaumberg DA (2007). Impact of dry eye syndrome on vision-related quality of life. Am J Ophthalmol.

[16] Pouyeh B, Viteri E, Feuer W, Lee DJ, Florez H, Fabian JA, Perez VL, Galor A (2012). Impact of ocular surface symptoms on quality of life in a United States veterans affairs population. Am J Ophthalmol.

[17] Li MY, Gong L (2011). [Progress of research on quality of life of dry eye patients]. Zhonghua Yan Ke Za Zhi.

[18] García-Catalán MR, Jerez-Olivera E, Benítez-Del-Castillo-Sánchez JM (2009). [Dry eye and quality of life]. Arch Soc Esp Oftalmol.

[19] Friedman NJ (2010). Impact of dry eye disease and treatment on quality of life. Curr Opin Ophthalmol.

[20] Pflugfelder SC, Tseng SC, Sanabria O, Kell H, Garcia CG, Felix C, Feuer W, Reis BL (1998). Evaluation of subjective assessments and objective diagnostic tests for diagnosing tear-film disorders known to cause ocular irritation. Cornea.

[21] Pflugfelder SC (2008). Prevalence, burden, and pharmacoeconomics of dry eye disease. Am J Manag Care.

[22] Yamada M (2009). [Review 38. Dry eye syndrome: concept, pathogenesis, and therapeutic modalities based on the new definition]. Nihon Ganka Gakkai Zasshi.

[23] Mizuno Y, Yamada M, Miyake Y, Dry Eye Survey Group of the National Hospital Organization of Japan (2010). Association between clinical diagnostic tests and health-related quality of life surveys in patients with dry eye syndrome. Jpn J Ophthalmol.

[24] Communications Usage Trend Survey.

[25] Collins M, Brown B, Bowman K, Carkeet A (1990). Workstation variables and visual discomfort associated with VDTs. Appl Ergon.

[26] Madhan MR (2009). Computer vision syndrome. Nurs J India.

[27] Blehm C, Vishnu S, Khattak A, Mitra S, Yee RW (2005). Computer vision syndrome: a review. Surv Ophthalmol.

[28] Rosenfield M (2011). Computer vision syndrome: a review of ocular causes and potential treatments. Ophthalmic Physiol Opt.

[29] Izquierdo JC, García M, Buxó C, Izquierdo NJ (2004). Factors leading to the Computer Vision Syndrome: an issue at the contemporary workplace. Bol Asoc Med P R.

[30] Ye Z, Abe Y, Kusano Y, Takamura N, Eida K, Takemoto T, Aoyagi K (2007). The influence of visual display terminal use on the physical and mental conditions of administrative staff in Japan. J Physiol Anthropol.

[31] Toda I, Fujishima H, Tsubota K (1993). Ocular fatigue is the major symptom of dry eye. Acta Ophthalmol (Copenh).

[32] Thomson WD (1998). Eye problems and visual display terminals--the facts and the fallacies. Ophthalmic Physiol Opt.

[33] Yaginuma Y, Yamada H, Nagai H (1990). Study of the relationship between lacrimation and blink in VDT work. Ergonomics.

[34] Nakaishi H, Yamada Y (1999). Abnormal tear dynamics and symptoms of eyestrain in operators of visual display terminals. Occup Environ Med.

[35] Miyake-Kashima M, Dogru M, Nojima T, Murase M, Matsumoto Y, Tsubota K (2005). The effect of antireflection film use on blink rate and asthenopic symptoms during visual display terminal work. Cornea.

[36] Uchino M, Nishiwaki Y, Michikawa T, Shirakawa K, Kuwahara E, Yamada M, Dogru M, Schaumberg DA, Kawakita T, Takebayashi T, Tsubota K (2011). Prevalence and risk factors of dry eye disease in Japan: Koumi study. Ophthalmology.

[37] Ishida R, Kojima T, Dogru M, Kaido M, Matsumoto Y, Tanaka M, Goto E, Tsubota K (2005). The application of a new continuous functional visual acuity measurement system in dry eye syndromes. Am J Ophthalmol.

[38] Nakamura S, Kinoshita S, Yokoi N, Ogawa Y, Shibuya M, Nakashima H, Hisamura R, Imada T, Imagawa T, Uehara M, Shibuya I, Dogru M, Ward S, Tsubota K (2010). Lacrimal hypofunction as a new mechanism of dry eye in visual display terminal users. PLoS One.

[39] Donker T, van Straten A, Marks I, Cuijpers P (2009). A brief Web-based screening questionnaire for common mental disorders: development and validation. J Med Internet Res.

[40] Ramo DE, Prochaska JJ (2012). Broad reach and targeted recruitment using Facebook for an online survey of young adult substance use. J Med Internet Res.

[41] Eysenbach G, Wyatt J (2002). Using the Internet for surveys and health research. J Med Internet Res.

[42] Wright JC, Meger GE (1962). A review of the Schirmer test for tear production. Arch Ophthalmol.

[43] Craig CL, Marshall AL, Sjöström M, Bauman AE, Booth ML, Ainsworth BE, Pratt M, Ekelund U, Yngve A, Sallis JF, Oja P (2003). International physical activity questionnaire: 12-country reliability and validity. Med Sci Sports Exerc.

[44] Murase N, Katsumura T, Ueda C, Inoue S, Shimomitsu T (2002). Validity and reliability of Japanese version of International Physical Activity Questionnaire. Kōsei no Shihyō.

